# Diagnosis and management guidelines for infantile epileptic spasms syndrome around the world: A scoping review and comparative study of international approaches

**DOI:** 10.1002/epi.70329

**Published:** 2026-06-11

**Authors:** Gozde Erdemir, Chethan K. Rao, Anne Francine Pino, Christina Briscoe, Debopam Samanta, Jo M. Wilmshurst, Sonal Bhatia, Jessica L. Carpenter, Christina Hoei‐Hansen, Puneet Jain, Tommy Stodberg, Robyn Whitney, Ryuki Matsuura, Karina Rosso Astorga, Keryma Acevedo Gallinato, Pratibha Singhi, Aristides Hadijinicolaou, Gia Melikishvili, Patricia Smeyers, Nicola Specchio, Stéphane Auvin, Pediatric Epilepsy Research Consortium Infantile Spasm Work Group and International Committee

**Affiliations:** ^1^ Department of Pediatrics, Division of Pediatric Neurology Penn State Health Milton S. Hershey Medical Center, Penn State College of Medicine Hershey Pennsylvania USA; ^2^ Department of Pediatrics University of Maryland School of Medicine Baltimore Maryland USA; ^3^ Pennsylvania State College of Medicine Hershey Pennsylvania USA; ^4^ Department of Pediatrics Boston Children's Hospital Boston Massachusetts USA; ^5^ Department of Pediatrics, Division of Pediatric Neurology University of Arkansas for Medical Sciences Little Rock Arkansas USA; ^6^ Department of Paediatric Neurology Red Cross War Memorial Children's Hospital, Neuroscience Institute, University of Cape Town Cape Town South Africa; ^7^ Department of Pediatrics University of Texas Southwestern Medical Center Dellas Texas USA; ^8^ Department of Pediatrics University Hospital Rigshospitalet Copenhagen Denmark; ^9^ Epilepsy Program, Division of Neurology, Department of Paediatrics Hospital for Sick Children, University of Toronto Toronto Ontario Canada; ^10^ Department of Pediatric Neurology Karolinska University Hospital Stockholm Sweden; ^11^ Department of Pediatrics McMaster University Hamilton Ontario Canada; ^12^ Division of Pediatric Epilepsy Center Saitama Children's Medical Center Saitama Japan; ^13^ Department of Pediatrics Hospital Carlos van Buren Valparaíso Chile; ^14^ Pediatric Neurology Unit, Division of Pediatrics School of Medicine, Pontificia Universidad Católica de Chile Santiago Chile; ^15^ Department of Pediatric Neurology Amrita Institute of Medical Sciences, Amrita Vishwa Vidyapeetham Faridabad India; ^16^ Department of Pediatrics and Neurosciences University of Montreal Montreal, Quebec Canada; ^17^ Mediclub Georgia Medical Center Tbilisi Georgia; ^18^ Department of Pediatric Neurology Hospital Universitario y Politécnico La Fe Valencia Spain; ^19^ Neurology, Epilepsy, and Movement Disorders Unit Bambino Gesù Children's Hospital, full member of European Reference Network EpiCARE, IRCCS Rome Italy; ^20^ University Hospitals KU Leuven Leuven Belgium; ^21^ AP‐HP, Pediatric Neurology Department Reference Center for Rare Epilepsies, member of European Reference Network EpiCARE, Hôpital Universitaire Robert Debré Paris France; ^22^ Institut Hospitalo‐Universitaire Robert‐Debré du Cerveau de L'enfant Paris France; ^23^ AP‐HP, Neurophysiology Department Reference Center for Rare Epilepsies, member of European Reference Network EpiCARE, Hôpital Universitaire Robert Debré Paris France; ^24^ Institut Universitaire de France Paris France

**Keywords:** health care disparities, infantile epileptic spasms syndrome, infantile spasm, pediatric epilepsy, treatment guideline

## Abstract

**Objective:**

Infantile epileptic spasms syndrome (IESS) is an epileptic encephalopathy requiring rapid diagnosis and treatment to optimize neurodevelopmental outcomes. Although multiple national and regional guidelines exist, recommendations vary. We conducted a comparative scoping review of international IESS guidelines to synthesize current recommendations, evaluate trends, and examine regional differences in diagnosis and treatment.

**Methods:**

Published guidelines were identified through MEDLINE, Embase, and Cochrane CENTRAL, using PRISMA‐ScR (Preferred Reporting Items of Systematic Reviews and Meta‐Analyses, extension for scoping reviews) methodology and PROSPERO (Prospective Register of Systematic Reviews) registration. Unpublished institutional protocols were obtained from pediatric neurologists worldwide. Extracted variables included diagnostic evaluation, treatment recommendations, dosing, response assessment, and monitoring. Descriptive analysis compared recommendations across regions and time periods.

**Results:**

Twenty‐eight guidelines were analyzed (16 published, 12 unpublished), including 15 from high‐income countries (HICs) and 11 from low‐ and middle‐income countries (LMICs). Electroencephalography was recommended in nearly all guidelines (27/28, 96%), whereas brain magnetic resonance imaging (20/28, 71%) and genetic testing (15/28, 54%) were less consistent. Vigabatrin was universally recommended as first‐line therapy for IESS‐associated tuberous sclerosis complex. Earlier guidelines (2004–2015) favored adrenocorticotropic hormone (ACTH), whereas recent guidelines (2016–2024) increasingly endorsed prednisolone and vigabatrin. Combination therapy emerged after 2017 (~25%). LMIC guidelines uniformly recommended prednisolone, whereas HIC guidelines more frequently endorsed ACTH, vigabatrin, and combination therapy. Treatment response was most often assessed at ~14 days.

**Significance:**

International IESS guidelines share core principles but demonstrate evolving practices and persistent global disparities. HIC and LMIC recommendation differences likely reflect variation in medication access, cost, and diagnostic resources. Harmonized international guidance may reduce disparities and improve equitable care. Further research is needed to develop standardized treatment protocols and explore novel therapeutic options to optimize outcomes for infants with IESS.

**Key points**

- IESS guidelines have evolved over 2 decades, with prednisolone replacing ACTH as the dominant first‐line agent and combination therapy emerging after 2017.
- Differences between HIC and LMIC guidelines may reflect structural barriers including cost, supply, and regulatory access rather than true disagreement on optimal therapy.
- Substantial variability persists globally in diagnostic workup, response assessment timing, monitoring protocols, and second‐line therapies.
- International consensus guidance with adaptable minimum standards is needed to reduce global disparities in IESS care.

## 1 INTRODUCTION

Infantile epileptic spasms syndrome (IESS) is the most common developmental and epileptic encephalopathy of infancy, with an estimated incidence of 30–40 per 100 000 live births.^1^ It requires rapid diagnosis and treatment compared to other infantile epilepsy syndromes.

Etiologies vary across geographic and economic settings. Structural etiologies, particularly perinatal brain injuries, predominate in low‐ and middle‐income countries (LMICs), and infectious causes contribute to IESS burden in endemic regions. Genetic etiologies are more frequently identified in high‐income countries (HICs), where testing is more accessible.^2^, ^3^, ^4^ Access to diagnostic tools such as video‐electroencephalography (EEG), magnetic resonance imaging (MRI), and genetic testing varies dramatically across settings, with particularly limited availability in LMICs, where approximately 80% of people with epilepsy reside.^5^ However, no standardized consensus exists regarding the extent of etiological evaluation in IESS, contributing to clinical practice variability.

In 2015, the International League Against Epilepsy (ILAE) published a task force report on infantile seizures management, followed by a Child Neurology Society quality measure set in 2016.^6^, ^7^ Both emphasize the importance of initiating standard therapy within 1 week of diagnosis, identifying adrenocorticotropic hormone (ACTH), high‐dose prednisolone, and vigabatrin as first‐line treatment options. Initiating standard therapy is important, as earlier intervention has been associated with a more favorable prognosis.^8^ However, important gaps remain, including optimal treatment sequencing, duration, and monitoring strategies.

Although evidence suggests that ACTH and high‐dose prednisolone may produce comparable response rates, there is no clear consensus on whether these therapies should be administered sequentially or in combination with vigabatrin.^9^ In addition, recommendations regarding second‐line therapies, ketogenic diet, surgery, and adverse effect monitoring remain inconsistent, with no existing standardized guidance.

Recent studies examining IESS management found that although 88% of centers recommended standard first‐line therapy, reflecting substantial consensus on core treatment, meaningful variation persisted in treatment sequencing, dosing protocols, and monitoring practices.^10^ It is likely that even greater differences exist in clinical practice globally. This variability stems from limited evidence for IESS treatment, particularly in the setting of randomized control trials (RCTs). A 2022 network meta‐analysis identified only 22 RCTs comparing first‐line treatments; however, the majority of RCTs have been small, with only two trials enrolling more than 100 patients.^9^, ^11^, ^12^ Treatment delay directly impacts neurodevelopmental outcomes.^13^ Yet, in many LMICs, the treatment gap remains unacceptably long, with many preventable IESS causes, including hypoxic–ischemic encephalopathy, neonatal hypoglycemia, and infectious etiologies.^2^, ^14^, ^15^ This prolonged treatment gap worsens outcomes and confounds assessment of treatment efficacy across settings. Understanding the global management of IESS is critical, given its profound neurological consequences. Despite current multiple national and regional guidelines, no comprehensive synthesis comparing international recommendations exists. In this scoping review, we synthesize the current evidence and summarize international IESS guidelines to provide a comprehensive overview of global practices, identify areas of consensus and variation, and highlight disparities in care.

## 2 MATERIALS AND METHODS

We conducted a scoping review of IESS guidelines using Embase, MEDLINE, and Cochrane CENTRAL. The protocol was registered on PROSPERO (Prospective Register of Systematic Reviews; CRD42024584217) and followed Preferred Reporting Items of Systematic Reviews and Meta‐Analyses, extension for scoping reviews (PRISMA‐ScR) guidelines.

### 2.1 Search strategy, study screening, and data extraction

The search strategy was developed by a pediatric neurologist (G.E.) and a medical librarian (Y.F.). The search was conducted in November 2024 across PubMed (1809–present), Embase (Embase.com, 1974–present), and Cochrane CENTRAL Register of Controlled Trials (Wiley). See Data S1 for search terms. Results were imported into Covidence, duplicates were removed, and results were screened independently by two authors (G.E. and C.K.R.). Full texts were assessed using predefined criteria, with discrepancies resolved by consensus.

Eligible studies included national or international guidelines providing diagnostic or treatment recommendations. Nonrelevant or duplicate studies were excluded.

Search results from each database were imported into systematic review software (Covidence [www.covidence.org]), and duplicates were removed. Two authors (G.E. and C.K.R.) independently screened the remaining citations by title and abstract. Full‐text articles were subsequently reviewed for relevance, eligibility, and quality by the same two authors using preestablished criteria. Discrepancies were resolved through discussion.

To address underrepresentation of non‐Western regions, unpublished guidelines were obtained through personal and professional pediatric neurology organizations, including ILAE and International Child Neurology Association, and translated when needed by local neurologists or by coauthor A.F.P. The number of individuals contacted and the response rate were not formally tracked.

Extracted variables included guideline characteristics, diagnostic evaluation, treatment strategies, dosing, response assessment, and monitoring. Variables were informed by prior Pediatric Epilepsy Research Consortium methodology.^10^ See Data S1 for additional details on data extraction.

### 2.2 Statistical analysis

Descriptive statistics summarized recommendations by region and used the World Bank income classification for guideline classification as either LMIC or HIC.

Comparisons between LMIC and HIC guidelines were performed for selected binary outcomes. Given small sample sizes, Fisher exact test (two‐sided) was used for exploratory comparisons, with effect sizes reported descriptively as proportions. All statistical comparisons were considered exploratory, given the descriptive intent of this review, and no adjustment was made for multiple comparisons. A sensitivity analysis was performed restricting the LMIC versus HIC comparison to national and international guidelines only, excluding institutional protocols, to assess whether conclusions were robust to differences in document type and development rigor.

## 3 RESULTS

### 3.1 Study selection

**FIGURE 1:**
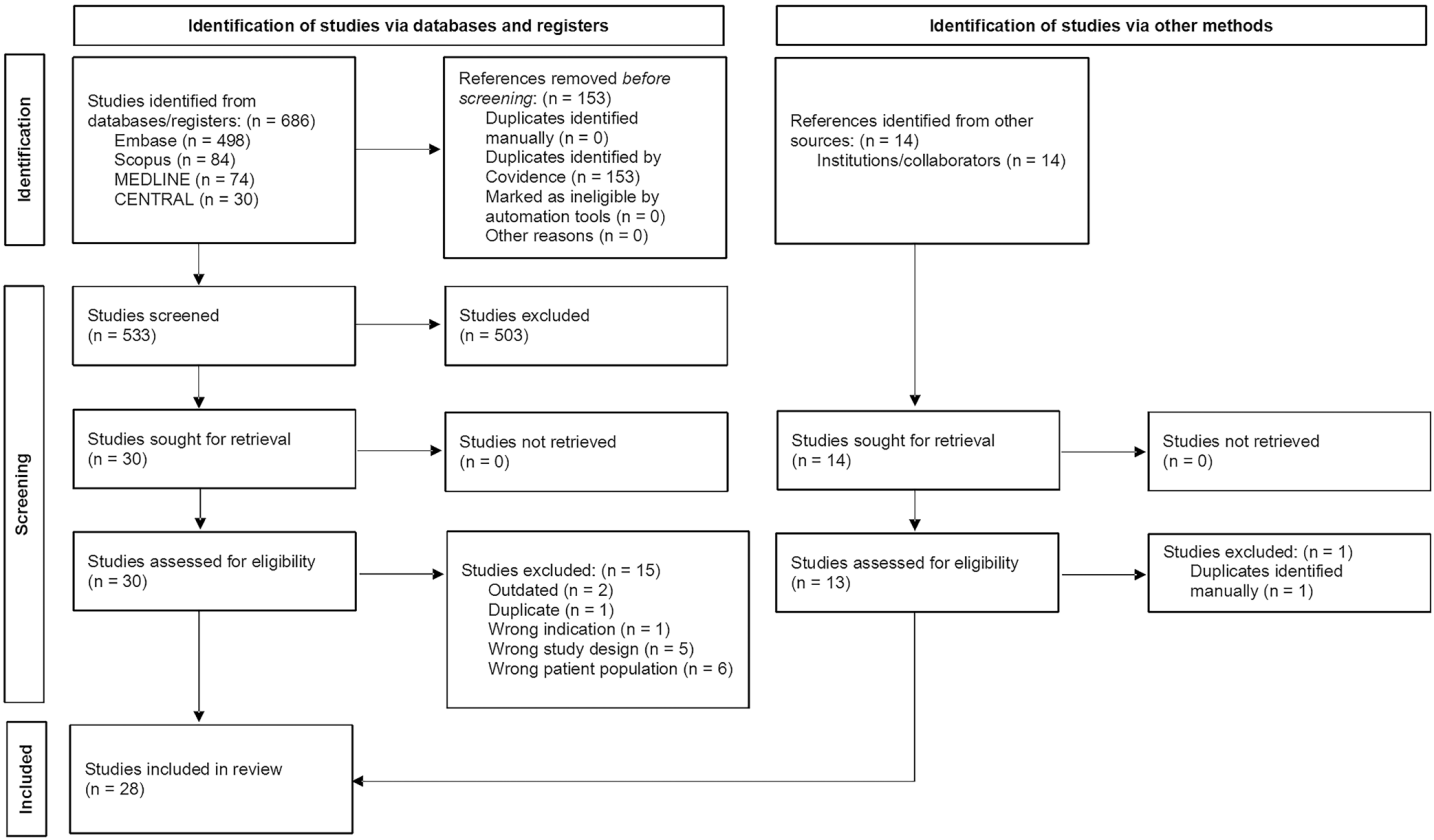
Preferred Reporting Items of Systematic Reviews and Meta‐Analyses, extension for scoping reviews diagram of guideline identification, screening, and inclusion.

A total of 686 studies were screened; 15 published guidelines met the inclusion criteria and were included in this review. Publication years ranged from 2004 to 2024. Additionally, 12 unpublished and one published guideline were obtained from pediatric neurologists worldwide. Together, 28 guidelines were analyzed (16 published, 12 unpublished). Figure 1 illustrates the search and selection process results according to PRISMA‐ScR methodology.

**FIGURE 2:**
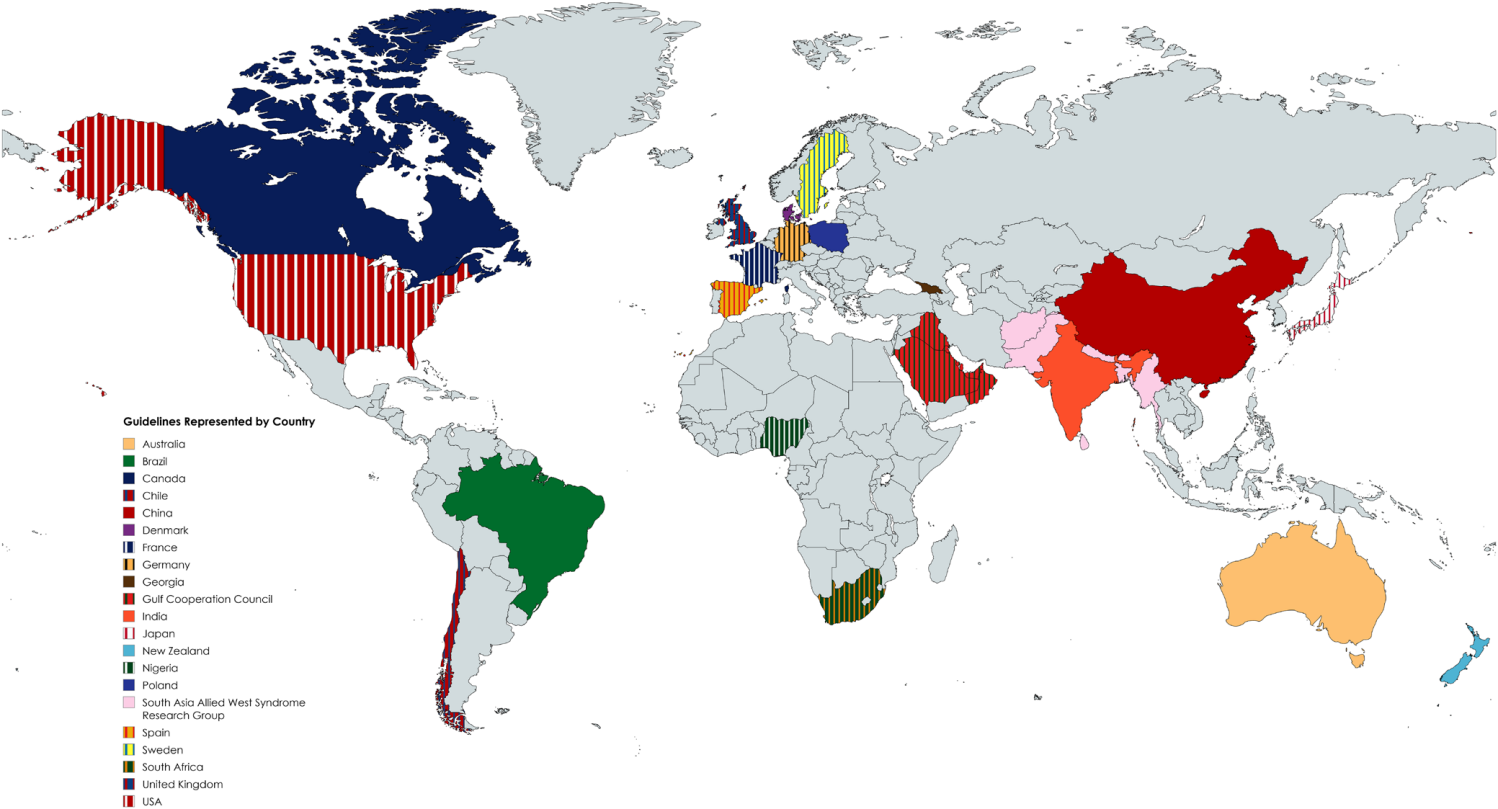
Guidelines represented by country.

Countries represented in the committees spanned major global regions, including Australia, Brazil, Canada, Chile, China, Denmark, France, Georgia, German‐speaking countries, Persian Gulf countries, India, Japan, New Zealand, Nigeria, Poland, South Africa, South Asia, Spain, Sweden, the United Kingdom, and the United States, represented in Figure 2.^6^, ^16^, ^17^, ^18^, ^19^, ^20^, ^21^, ^22^, ^23^, ^24^, ^25^, ^26^, ^27^, ^28^, ^29^, ^30^, ^31^, ^32^, ^33^, ^34^, ^35^, ^36^, ^37^, ^38^, ^39^, ^40^, ^41^, ^42^ Overall guidelines included eight (29%) institutional, 14 (50%) national, and six (21%) international in scope. Four (14%) specifically focused on management during the COVID‐19 pandemic, and one guideline addressed specific management in LMICs.^41^ Eight (29%) were nonspecific to IESS but addressed the management of pediatric or infantile epileptic syndromes and included IESS‐relevant sections. Some guidelines only focused on IESS treatment. One consensus was for case definitions and outcome measures.^28^ Included documents varied in type, purpose, and development process. National and international guidelines generally underwent structured development involving multidisciplinary expert panels and formal endorsement by professional societies, whereas institutional protocols function primarily as practical clinical manuals based on local expert consensus. Unpublished documents reflect real‐world clinical practice but may not represent formally endorsed guidance. These distinctions should be considered when interpreting the variability in recommendations described in subsequent sections.

In accordance with the World Bank income classification, we categorized guidelines as 11 (39%) LMIC and 15 (54%) HIC. Two (7%) guidelines were multinational and nonspecific and therefore were not included in comparative analysis of regional income.

Table 1 provides a summary of included guideline characteristics.

**TABLE 1:** Characteristics of included IESS guidelines.

Year	Author	Country; association	Scope	Guideline type	Published
2004	Lux et al.	West Delphi Group	Case definitions and outcome measures	International	Yes
2004	Mackay et al.	USA; AAN/CNS	Diagnosis and management	National	Yes
2009	Expert Committee on Pediatric Epilepsy	India; Indian Academy of Pediatrics	Diagnosis and management of childhood epilepsy^a^	National	Yes
2010	Pellock et al.	USA	Diagnosis and management	National	Yes
2015	Dunin‐Wąsowicz et al.	Poland	Management of pediatric epilepsy^a^	National	Yes
2015	Wilmshurst et al.	ILAE	Diagnosis and management of pediatric epilepsy^a^	International	Yes
2017	Dozières‐Puyravel & Auvin	France	Diagnosis and management	Institutional	No
2018	Based on Raga et al.	South Africa	Diagnosis and management for pediatric epilepsy^a^	Institutional	No
2020	Boyd et al.	Canada	Diagnosis and management	Institutional	No
2020	Chen et al.	China	Management during COVID‐19	National	Yes
2020	Grinspan et al.	USA	Management during COVID‐19	National	Yes
2020	Sahu et al.	South Asian Allied West Syndrome Research Group	Management during COVID‐19	International	Yes
2020	Royal Children's Hospital Melbourne	Australia	Diagnosis and management	Institutional	No
2021	Departament de Salut València La Fe	Spain	Diagnosis and management	Institutional	No
2021	Igwe et al.	Nigeria	Diagnosis and management of pediatric epilepsy^a^	National	No
2021	Madaan et al.	South Asia Allied West Syndrome Research Group	Management during COVID‐19	International	Yes
2021	Sharma et al.	India; Indian Epilepsy Society	Diagnosis and management	National	Yes
2022	Davis et al.	New Zealand	Diagnosis and management for pediatric epilepsy^a^	National	No
2022	Kasradze et al.	Georgia	Diagnosis and management for pediatric epilepsy^a^	National	No
2022	National Institute for Health and Care Excellence	United Kingdom	Diagnosis and management for pediatric epilepsy^a^	International	No
2022	Ramantani et al.	German‐Speaking Society of Neuropediatrics	Diagnosis and management	National	Yes
2022	Sampaio et al.	Brazil	Diagnosis and management	National	Yes
2023	Isaksson et al.	Sweden; Karolinska University Hospital	Diagnosis and management	Institutional	No
2023	Matsuura et al.	Japan; Saitama Children's Medical Center	Diagnosis and management	Institutional	Yes
2024	Acevedo et al.	Chile	Diagnosis and management	National	No
2024	Bashiri et al.	Gulf Cooperation Council countries	Diagnosis and management	International	Yes
2024	Copenhagen University, national protocol	Denmark; Danish Pediatric Society	Diagnosis and management	Institutional	No
2024	Sahu et al.	South Asia Allied West Syndrome Research Group	Diagnosis and management	International	Yes

*Note*: Twenty‐eight guidelines met inclusion criteria.

Abbreviations: AAN/CNS, American Academy of Neurology/Child Neurology Society; IESS, infantile epileptic spasms syndrome; ILAE, International League Against Epilepsy.

^a^ Contains section for IESS.

### 3.2 Diagnostic evaluation recommendations

#### 3.2.1 EEG monitoring

Nearly all guidelines (27/28, 96%) recommended obtaining a diagnostic EEG prior to initiating treatment; the single guideline that did not comment on EEG lacked a diagnostic workup section.^25^ Fourteen (50%) guidelines recommended inpatient EEG monitoring, whereas 11 (39%) endorsed outpatient EEG, most often when inpatient studies were unavailable or during the COVID‐19 pandemic. Video‐EEG was most commonly recommended, with inclusion of at least one sleep–wake cycle.

LMIC guidelines universally recommended EEG prior to treatment but emphasized that therapy should not be delayed by limited access. Several noted that outpatient EEG with sleep could be an acceptable alternative when inpatient EEG was unavailable.

All four (14%) COVID‐19‐focused management guidelines recommended pretreatment EEG.

Eighteen (64%) guidelines commented that hypsarrhythmia on EEG was highly suggestive of IESS; however, the general consensus agreed that its presence was not required for ultimate diagnosis.

Regarding EEG monitoring duration, five (18%) guidelines recommended at least 24 h, provided that a sleep–wake cycle was captured. Four (14%) guidelines explicitly recommended at least one full sleep–wake cycle, whereas three (11%) advised a minimum study duration of 4 h. The remaining 16 (57%) guidelines did not mention EEG duration specifics.

#### 3.2.2 Neuroimaging

Although not all guidelines provided diagnostic recommendations, 20 (71%) guidelines endorsed obtaining a brain MRI during initial diagnostic evaluation.

However, if not immediately available, it should be obtained within 1–3 days and is especially indicated with onset before age 2 years, clinical/EEG features of focal seizures, and drug‐resistant spasms.^23^ Guidelines emphasized that treatment should not be delayed while awaiting MRI results. If initial MRI is normal, then one guideline recommended a repeat MRI at approximately 6 months, and especially after 24 months, to detect cortical dysplasia missed on early imaging and features suggestive of genetic/metabolic disease.^21^ Commonly mentioned MRI modalities were 3‐T epilepsy‐protocol, with core sequences including three‐dimensional (3D) T1, 3D fluid‐attenuated inversion recovery (FLAIR), high‐resolution coronal T2, and optional diffusion‐weighted imaging.^21^, ^26^, ^27^ One guideline commented that consideration in patients less than 1 year old should take place, as FLAIR and 3D T1 are less sensitive, and preference should be given high‐resolution T2 sequences in multiple planes.^26^ Limited MRI availability was reported as a major barrier in low‐resource settings.^26^

#### 3.2.3 Genetic and metabolic testing

Fifteen (54%) guidelines endorsed genetic testing, especially in treatment‐resistant cases. Where specified, seven (25%) guidelines, published between 2021 and 2024, recommended whole exome sequencing (WES) or whole genome sequencing (WGS). First‐line testing strategies include chromosomal microarray, high‐resolution karyotype, and fluorescence in situ hybridization in children with dysmorphism. If first‐line testing is negative, one guideline recommended an epileptic encephalopathy gene panel.^36^ Then, WES, preferably trio testing for variant segregation, and WGS are recommended.

Twelve (43%) guidelines recommended including metabolic testing during initial workup, especially when etiology is unclear or prompted by specific clinical concerns.^21^ The most common recommended first‐line testing included serum glucose, basic hematologic screening, liver function tests, ammonia, urinalysis, pH, arterial blood gases, and serum electrolytes. The Persian Gulf region guideline recommended serum amino acid, lactate, biotinidase and gas chromatography–mass spectrometry urine test as essential basic metabolic investigations.^16^ Cerebrospinal fluid testing was not frequently included in first‐line evaluation, rather only when GLUT1 deficiency is suspected. Several guidelines cited the following clinical features as indications for metabolic‐focused investigations: movement disorders, failure to thrive, systemic features (e.g., liver disease, dysmorphism), MRI suggestive of mitochondrial/white matter disease, and refractory spasms without structural cause or with poor response to conventional treatment.

Table 2 provides a summary of diagnostic evaluation recommendations.

**TABLE 2:** Summary of diagnostic evaluation recommendations across IESS guidelines.

Diagnostic modality	Recommendation	Guidelines endorsing, *N* = 28, *n* (%)	Notes
EEG	EEG prior to treatment	27 (96%)	One guideline did not comment^25^; recommended by all COVID‐19‐specific guidelines^20^, ^22^, ^23^, ^24^
	Inpatient EEG monitoring	14 (50%)	Preferred when available; video‐EEG most commonly recommended
	Outpatient EEG monitoring	11 (39%)	Suggested when inpatient EEG unavailable or during COVID‐19 pandemic
	Hypsarrhythmia noted	18 (64%)	Highly suggestive of IESS but not required for diagnosis
EEG duration	≥24 h or until sleep–wake cycle captured	5 (18%)	
	At least one full sleep–wake cycle	4 (14%)	
	Minimum of 4 h	3 (11%)	
	Duration not specified	16 (57%)	
Neuroimaging	Brain MRI in initial evaluation	20 (71%)	
	Specified imaging considerations	1 (4%)	
Genetic testing	Genetic testing recommended	15 (54%)	Particularly for treatment‐resistant or unknown etiology spasms
	WES or WGS specified	7 (25%)	Endorsed by guidelines published between 2021 and 2024
Metabolic testing	Included in initial evaluation	12 (43%)	

*Note*: Diagnostic evaluation recommendations across 28 clinical guidelines for IESS. Percentages represent the proportion of guidelines endorsing each recommendation.

Abbreviations: EEG, electroencephalography; IESS, infantile epileptic spasms syndrome; MRI, magnetic resonance imaging; WES, whole exome sequencing; WGS, whole genome sequencing.

### 3.3 First‐line treatment recommendations

Table 3 provides a summary of treatment and management recommendations.

**TABLE 3:** Summary of treatment and management recommendations across IESS guidelines.

Treatment domain	Recommendation	Guidelines endorsing, *n* (%)	Notes
Treatment	Provided treatment recommendations	26/28 (93%)	Two guidelines did not provide treatment recommendations^17^, ^28^
TSC‐associated IESS	Vigabatrin first‐line	26/26 (100%)	
First‐line agents	Prednisolone	20/26 (77%)	
	Vigabatrin	19/26 (73%)	
	ACTH	17/26 (65%)	
Combination therapy	Hormonal therapy + vigabatrin	7/26 (27%)	Emerged after 2017
Temporal trends	Early guidelines recommending ACTH (2004–2015)	6/6 (100%)	Prednisolone also recommended in 2/6 (33%)
	Later guidelines recommending prednisolone (2016–2024)	18/20 (90%)	ACTH in 11/20 (55%), vigabatrin in 13/20 (65%)
Medication dosing protocols	ACTH	11/28 (39%)	Regimens ranged from 20 to 150 IU/day or 40–60 IU every other day
	Prednisolone	19/28 (68%)	Most common: 4–8 mg/kg/day (maximum 60 mg/day)
	Vigabatrin	14/28 (50%)	Common doses: 50–150 mg/kg/day
	Steroid tapering regimen described	14/28 (50%)	
Response assessment timing	Assessment at ~14 days after treatment initiation	14/28 (50%)	
	Early assessment at 5–10 days	7/28 (25%)	
Response timing	Assess at 14 days	14/28 (50%)	Most common recommendation
	Early assessment (5–10 days)	7/28 (25%)	
	Clinical + EEG confirmation of response	20/28 (71%)	Cessation of spasms and improvement of hypsarrhythmia
Second‐Line and adjunctive treatments	Ketogenic diet recommended	21/28 (75%)	Typically after failure of first‐line therapy
	Alternative antiseizure medications	10/28 (36%)	Valproate, topiramate, clobazam, vitamin therapies
	Epilepsy surgery mentioned	16/28 (57%)	Mainly for structural etiologies or refractory spasms
Safety monitoring and supportive care	Side effect monitoring protocols included	20/28 (71%)	
	Blood pressure monitoring	18/28 (64%)	Recommended during steroid therapy
	Glucose monitoring	14/28 (50%)	Recommended during steroid therapy
	Ophthalmologic monitoring	11/28 (39%)	Recommended during vigabatrin therapy
	Infection prophylaxis guidance	2/28 (7%)	Trimethoprim–sulfamethoxazole for PJP prevention
	Vaccination guidance included	7/28 (25%)	Avoid live vaccines during steroid therapy
	Parental education recommended	14/28 (50%)	Education on treatment, adverse effects, and supportive care

*Note*: Percentages represent the proportion of guidelines endorsing each recommendation.

Abbreviations: ACTH, adrenocorticotropic hormone; EEG, electroencephalographic; IESS, infantile epileptic spasms syndrome; PJP, *Pneumocystis jirovecii* pneumonia; TSC, tuberous sclerosis complex.

Among the 28 identified guidelines, 26 (93%) provided specific treatment recommendations; two (7%) did not comment on treatment.^17^, ^28^

For IESS secondary to tuberous sclerosis complex (TSC), vigabatrin was universally recommended as first‐line therapy.

For non‐TSC related IESS, most guidelines (22/26, 84%) offered multiple first‐line options, mainly ACTH, oral steroids (prednisolone) and/or vigabatrin. Four guidelines recommended a single first‐line therapy: prednisolone^33^, ^37^, ^40^ or vigabatrin.^30^ One guideline proposed etiology‐specific treatment: ACTH or prednisolone for nonstructural etiologies, and ACTH ± vigabatrin for structural etiologies.^31^ One guideline additionally listed valproic acid as a first‐line option, whereas another included pyridoxal alongside ACTH or vigabatrin.^25^, ^35^

Across the 26 guidelines, 20 (77%) recommended prednisolone, 19 (73%) vigabatrin, and 17 (65%) ACTH, either as first‐line monotherapy or as part of combination therapy.

Seven (27%) guidelines endorsed upfront combination therapy with ACTH or prednisolone and vigabatrin.

Regarding temporal trends, early guidelines (2004–2015, *n* = 6) uniformly recommended ACTH (6/6, 100%), whereas prednisolone was cited in two of six (33%). Later guidelines (2016–2024, *n* = 20) offered greater flexibility, with prednisolone increasingly dominant (18/20, 90%), ACTH decreasing to 11 of 20 (55%), and vigabatrin in 14 of 20 (70%).

Combination therapy emerged after 2017 and became increasingly common as first‐line treatment (7/26, 27%).

Pandemic‐era guidelines (2020–2021) highlighted prednisolone to facilitate outpatient treatment and reduce hospital visits.

Regarding LMICs versus HICs, guidelines from HICs (North America, Western Europe, Japan, Australia, New Zealand) more frequently endorsed ACTH and vigabatrin and were more likely to recommend combination therapy. Among HIC guidelines (*n* = 15), 10 of 15 (67%) endorsed ACTH, nine of 15 (60%) endorsed prednisolone, 12 of 15 (80%) endorsed vigabatrin, and four of 15 (27%) recommended combination therapy.^16^, ^19^, ^35^, ^36^, ^42^ In contrast, LMIC guidelines (*n* = 10), namely from South Asia, Africa, Latin America, and parts of Eastern Europe, prioritized prednisolone as first‐line therapy (10/10, 100%), whereas ACTH was recommended in seven of 10 (70%), vigabatrin in six of 10 (60%), and combination therapy in only two of 10 (20%).^31^, ^34^

Exploratory comparisons confirmed that LMIC guidelines more frequently recommended prednisolone compared to HIC guidelines (*p* = .051), whereas recommendations for ACTH, vigabatrin, and combination therapy were statistically similar between groups.

In a sensitivity analysis restricted to national and international guidelines only, excluding eight institutional protocols, the LMIC group comprised nine guidelines and the HIC group comprised eight guidelines. Findings were directionally consistent with the primary analysis; LMIC guidelines continued to more frequently recommend prednisolone compared to HIC guidelines (100% vs. 62%, *p* = .082), whereas recommendations for ACTH (67% vs. 75%, *p* = 1.000), vigabatrin (67% vs. 88%, *p* = .576), and combination therapy (22% vs. 38%, *p* = .620) remained statistically similar between groups, suggesting that the inclusion of institutional protocols did not materially influence the primary findings.

Several LMIC guidelines explicitly cited limitations in medication availability, cost, and regulatory approval, and proposed stepwise or alternative approaches when ACTH or vigabatrin were inaccessible.^17^, ^18^, ^20^, ^24^ HIC guidelines more frequently referenced RCT evidence and emphasized early intensive or combination therapy.

Tables 4 and 5 compare LMIC and HIC guideline treatment recommendations.

**TABLE 4:** Treatment recommendations across international guidelines for infantile epileptic spasms syndrome: HICs versus LMICs.

Year	Guideline author	Country/region	Income classification	ACTH	Prednisolone	Vigabatrin	Combination therapy
2004	Mackay et al.	USA	HIC	Yes	No	Yes	No
2009	Expert Committee on Pediatric Epilepsy	India	LMIC	Yes	Yes	No	No
2010	Pellock et al.	USA	HIC	Yes	No	Yes	No
2015	Dunin‐Wąsowicz et al.	Poland	HIC	Yes	No	Yes	No
2017	Dozières‐Puyravel & Auvin	France	HIC	No	Yes	Yes	Yes
2018	Based on Raga et al.	South Africa	LMIC	Yes	Yes	Yes	No
2020	Boyd et al.	Canada	HIC	Yes	Yes	Yes	No
2020	Chen et al.	China	LMIC	No	Yes	Yes	No
2020	Grinspan et al.	USA	HIC	Yes	Yes	Yes	No
2020	Sahu et al.	South Asia	LMIC	Yes	Yes	Yes	No
2020	Royal Children's Hospital Melbourne	Australia	HIC	No	Yes	No	No
2021	Departament de Salut València La Fe	Spain	HIC	Yes	No	Yes	Yes
2021	Igwe et al.	Nigeria	LMIC	No	Yes	No	No
2021	Madaan et al.	South Asia	LMIC	Yes	Yes	Yes	No
2021	Sharma et al.	India	LMIC	Yes	Yes	No	No
2022	Davis et al.	New Zealand	HIC	No	Yes	No	No
2022	Kasradze et al.	Georgia	LMIC	No	Yes	Yes	Yes
2022	National Institute for Health and Care Excellence	United Kingdom	HIC	No	Yes	Yes	Yes
2022	Ramantani et al.	Germany	HIC	Yes	Yes	Yes	Yes
2022	Sampaio et al.	Brazil	LMIC	Yes	Yes	Yes	No
2023	Isaksson et al.	Sweden	HIC	Yes	Yes	No	No
2023	Matsuura et al.	Japan	HIC	Yes	No	Yes	No
2024	Acevedo et al.	Chile	LMIC	Yes	Yes	Yes	Yes
2024	Bashiri et al.	GCC Countries	HIC	Yes	Yes	Yes	Yes
2024	Copenhagen University, National Protocol	Denmark	HIC	No	No	Yes	No

Abbreviations: ACTH, adrenocorticotropic hormone; GCC, Gulf Cooperation Council; HIC, high‐income country; LMIC, low‐ and middle‐income country.

**TABLE 5:** Comparison of first‐line treatment recommendations for infantile epileptic spasms syndrome across income groups and publication periods.

Guideline scope	Prednisolone recommended	ACTH recommended	Vigabatrin recommended	Combination therapy recommended
Income group comparison				
LMIC guidelines [*N* = 10], *n* (%)	10 (100%)	7 (70%)	6 (60%)	2 (20%)
HIC guidelines [*N* = 15], *n* (%)	9 (60%)	10 (67%)	12 (80%)	4 (27%)
*p*	.051	1.000	.378	1.000
Publication period comparison				
Early guidelines [2004–2015, *N* = 6], *n* (%)	2 (33%)	6 (100%)	–	0 (0%)
Later guidelines [2016–2024, *N* = 20], *n* (%)	18 (90%)	11 (55%)	–	7 (35%)
*p*	.013^a^	.070	–	.130

*Note*: Guidelines from LMICs more frequently recommended prednisolone as first‐line therapy compared with HIC guidelines (100% vs. 60%, p = .051), whereas recommendations for ACTH, vigabatrin, and combination therapy were similar between groups. Over time, later guidelines (2016–2024) more frequently recommended prednisolone than earlier guidelines (2004–2015; 90% vs. 33%, *p* = .013), whereas ACTH recommendations decreased and combination therapy appeared only in later guidelines. Probability values were calculated using Fisher exact test.

Abbreviations: ACTH, adrenocorticotropic hormone; HIC, high‐income country; LMIC, low‐ and middle‐income country.

^a^ Statistically significant.

### 3.4 Timing of response assessment

Fourteen (50%) guidelines recommended evaluation 14 days after initiating treatment, advising clinicians to consider whether to continue, taper, or change therapy.

Seven (25%) guidelines recommended earlier assessment between 5 and 10 days.^17^, ^18^, ^20^, ^26^, ^30^, ^31^, ^34^

Response criteria recommendations were consistent among most guidelines. Twenty (71%) recommended using both clinical cessation of spasms and hypsarrhythmia resolution or marked improvement on EEG to evaluate for adequate treatment response. In LMICs, EEG confirmation is preferred; however, the decision may be individualized based on feasibility and EEG access. Outpatient EEG with at least one sleep–wake cycle is preferred.^23^, ^24^

### 3.5 Second‐line and other treatment options

The ketogenic diet was widely recommended, with 21 (75%) guidelines supporting its use after first‐line therapy fails. Indications included treatment‐resistant epileptic spasms, contraindications to steroids or vigabatrin, and certain metabolic or genetic etiologies (e.g., pyridoxine‐dependent epilepsy, pyridoxal‐5‐phosphate deficiency, nonketotic hyperglycinemia and *TSC1* and *TSC2* mutations, and *SCN2A* pathogenic variants). Ten (36%) guidelines endorsed agents such as valproate, topiramate, clobazam, and vitamin supplementation (e.g., vitamin B6, biotinidase, folic acid) in cases with limited to no response to first‐line treatments.^31^, ^38^

Sixteen (57%) guidelines discussed epilepsy surgery, typically recommended with structural etiologies or treatment‐resistant epileptic spasms. Timing for surgical evaluation was not mentioned; however, early recognition of potential candidates was recommended, especially in those with identifiable focal, structural, and operable lesions. Utilization of epilepsy surgery varied regionally; for example, it was reported as less common in Poland compared with other EU countries and the United States and not easily accessible in Nigeria.^25^, ^37^

### 3.6 Dosing protocols

For ACTH, regimens ranged from 40 to 60 IU intramuscularly every other day (4/28, 14%), to 150 IU daily (3/28, 11%). Less common approaches included ACTH 20–30 IU/day (2/28, 7%), tetracosactide depot (synthetic ACTH analog) 1 mg/mL every other day (1/28, 4%), and ACTH .001 mg/kg/day tapered over 3 weeks (1/28, 4%).

For prednisolone, five (18%) guidelines recommended 10 mg four times daily, whereas 14 (50%) recommended 4–8 mg/kg/day, with a maximum of 60 mg/day. Tapering schedules generally spanned 2–6 weeks.

For vigabatrin, six (21%) guidelines recommended 50 mg/kg/day, whereas eight (29%) endorsed 100–150 mg/kg/day. The maximum commonly cited dose was 150 mg/kg/day. There was no consistent recommendation for a tapering schedule; however, one guideline recommended an incremental rate of 50 mg/kg/day on day 1, 100 mg/kg/day on day 2, 125 mg/kg/day on day 3, and 150 mg/kg/day on day 4.^36^

### 3.7 Side effect monitoring and supportive care

#### 3.7.1 Monitoring

Twenty (71%) guidelines recommended formal monitoring protocols, with baseline laboratory studies mentioned by 13 (46%). Eighteen (64%) included blood pressure monitoring, and 14 (50%) included glucose checks during steroid therapy. Eleven (39%) specifically advised ophthalmologic monitoring with vigabatrin.

#### 3.7.2 Infection prophylaxis

Only two (7%) guidelines recommended prophylaxis against *Pneumocystis jirovecii* pneumonia during steroid use, namely with trimethoprim/sulfamethoxazole.^34^, ^36^ Six (21%) recommended a "steroid sick plan." These recommendations were not regionally specific.

#### 3.7.3 Vaccination

Seven (25%) guidelines addressed vaccination, generally recommending continuing routine immunizations but avoiding live vaccines (e.g., measles–mumps–rubella, varicella) during prolonged steroid therapy. One guideline specified avoiding live vaccines 4 weeks prior to and after corticosteroid or ACTH therapy. Additionally, inactivated vaccines, although immune response may be limited, can be administered until 1 week prior to and after therapy.^19^

#### 3.7.4 Parent education

Fourteen (50%) guidelines emphasized parental education regarding treatment, side effects, and supportive care.

## 4 DISCUSSION

This is the first scoping review to examine national and institutional IESS guidelines worldwide. The incorporation of published and unpublished guidelines offers real‐world clinical guidance that may not otherwise be accessible through traditional literature searches. A central finding is the marked underrepresentation of LMICs and regions outside Europe and North America in published literature, despite these regions comprising the majority of the global population. The inclusion of unpublished guidelines partially mitigated this imbalance and revealed substantial practice diversity that is not fully reflected in current literature. Included documents varied in scope and development rigor; international and national guidelines generally underwent structured, evidence‐based development processes, whereas institutional protocols typically serve as practical clinical manuals developed by local expert consensus. Unpublished documents reflect real‐world practice but may not represent formally endorsed guidance. These distinctions are important for contextualizing the heterogeneity of recommendations described below.

### 4.1 Diagnostic evaluation: Consensus with pragmatic adaptations

There was a strong consensus across continents on the need for EEG prior to treatment. However, substantial variability persists in recommendations regarding inpatient versus outpatient monitoring and the duration of EEG studies, differences that likely reflect disparities in resource availability. A recent study suggested that a single sleep–wake cycle (median duration = 107.5 min) demonstrates 84% sensitivity and 100% specificity for detecting IESS, potentially reducing median EEG recording time by approximately 800 min compared to full studies.^43^ For capturing epileptic spasms specifically, 4–5 h of video‐EEG monitoring appears optimal, with average time to first spasm capture of 188 min.^44^

MRI and genetic testing are widely recommended, but several LMIC guidelines make exceptions based on cost or feasibility, illustrating a pragmatic approach in resource‐constrained settings. Genetic evaluation has become more prominent in recent guidelines. Increased support for WES and WGS reflects advances in genomic technology and higher diagnostic yield in spasms of unknown etiology. First‐tier tests, including chromosomal microarray and targeted gene panels, remain appropriate in selected settings.

Overall, these recommendations point to the emergence of a global diagnostic standard while simultaneously highlighting structural access inequities that continue to shape diagnostic pathways.

### 4.2 Treatment

Across all settings, vigabatrin was consistently recommended for IESS secondary to TSC. For other etiologies, ACTH, high‐dose prednisolone, and vigabatrin were uniformly identified as core first‐line treatments. For all first‐line options, treatment duration differences are modest, but tapering schedules vary widely, reflecting limited comparative evidence.

The temporal shift from uniform ACTH recommendation in early guidelines (2004–2015) to increasing prednisolone dominance in recent guidelines (2016–2024) reflects evolving evidence and practical considerations. In 2012, the American Academy of Neurology recommended ACTH or vigabatrin as first‐line treatments, although this guideline has since been retired.^45^ Subsequent studies supported broader treatment options; the National Infantile Spasms Consortium found no significant difference between ACTH and oral steroids (46% vs. 44% freedom from treatment failure at 60 days), validating both as equivalent first‐line choices.^46^ Most guidelines recommended a 2–4‐week high‐dose steroid course followed by a taper over 2–6 weeks, adjusted according to clinical and EEG response, an area of relative consensus despite variability in agent selection and dosing.

Clinical implementation of ACTH also varies widely due to its limited availability among LMICs and HICs alike. Cost serves as the most substantial barrier, with additional limiting factors including supply limitations and potential adverse effects.^47^

The ICISS trial demonstrated that combination hormonal therapy plus vigabatrin achieved higher rates of spasm cessation than hormonal therapy alone, supporting the 27% of guidelines that recommend upfront combination therapy.^48^ The emergence of combination therapy recommendations exclusively after 2017 directly parallels publication of this trial. However, long‐term follow‐up of the ICISS cohort did not demonstrate significant differences in epilepsy outcomes or neurodevelopmental status at 4 years of age, which may partially explain why sequential therapy remains the most commonly used approach in practice.^9^, ^49^

Additionally, pandemic‐era guidelines explicitly prioritized prednisolone to facilitate outpatient management, highlighting how practical implementation considerations influence guideline development beyond pure efficacy data.

The divergence between HIC and LMIC guidelines may reflect documented barriers in medication access, cost, and regulatory approval. The marked increase in ACTH cost over the past 2 decades alongside decreasing prednisolone cost likely explains the universal adoption of prednisolone in LMIC guidelines.^50^ Similarly, lower rates of vigabatrin recommendations in LMICs, along with explicit statements regarding regulatory unavailability in several guidelines, reflect access constraints rather than differing interpretations of efficacy evidence. Vigabatrin was more commonly recommended in European guidelines, consistent with earlier regulatory approval and broader regional availability. These observations suggest that improving equitable access to existing medications and diagnostic tools may be the most actionable path toward reducing global disparities in IESS outcomes.

Second‐line treatment recommendations were highly variable. Although some guidelines suggested valproate, topiramate, or clobazam, evidence supporting their efficacy in IESS is limited. The ketogenic diet was widely endorsed after first‐line failure across regions, although the timing of initiation was unclear. Surgery was recognized as an important option for treatment‐resistant IESS or structural etiologies, but was less emphasized in LMIC guidelines, most likely due to resource limitations.

### 4.3 Response assessment, outcome measures, and supportive care

Most guidelines recommended early assessment of treatment response, typically at approximately 14 days. This may reflect the timepoint used in most RCTs, including the landmark UKISS and ICISS trials.^9^, ^51^ This standardized assessment window has become conventional in clinical practice and research, enabling cross‐study comparisons and informing treatment escalation decisions. However, the 14‐day timepoint may be longer than necessary for identifying treatment responders. Recent prospective data from the National Infantile Spasms Consortium demonstrated that 81% of responders achieve clinical remission within the first week of treatment, with median time to response of 3–4 days across all standard therapies.^52^ Real‐world data from a French cohort similarly found that 7 days is sufficient to assess treatment response, enabling rapid addition of sequential therapy for nonresponders.^53^

These findings support the 25% of guidelines recommending earlier assessment at 5–10 days, potentially enabling more rapid sequential therapy and minimizing treatment delay.

Monitoring guidance is similarly variable, especially regarding blood pressure, glucose, infection prophylaxis, and vigabatrin ophthalmologic surveillance. Recent US survey data show that only 79% of institutional protocols mention gastrointestinal prophylaxis and blood pressure monitoring, highlighting variability even in high‐resource settings.^10^

### 4.4 Implications, strengths, and future directions

These findings highlight both progress and persistent gaps in global IESS guideline development. High‐quality evidence for IESS management remains limited. Despite shared therapeutic foundations in management, this review demonstrates that substantial global variation persists in diagnostic evaluation, treatment sequencing, monitoring strategies, and supportive care. These differences reflect evolving evidence and regional disparities in health system infrastructure, medication availability, and access to diagnostic resources. Aligning with the goals of the World Health Organization Intersectoral Global Action Plan on Epilepsy and Other Neurological Disorders, these findings emphasize the need for global minimum standards that ensure timely diagnosis and treatment while remaining flexible enough to fit different resource settings.^54^

These findings carry direct clinical and policy implications. Clinicians in all settings should initiate treatment within 1 week of diagnosis using ACTH, prednisolone, or vigabatrin, consistent with the consensus across guidelines. In resource‐limited settings, high‐dose prednisolone represents an evidence‐supported, cost‐effective first‐line option. Earlier response assessment at 5–10 days warrants consideration, given emerging evidence that most responders achieve remission within the first week, enabling more rapid escalation for nonresponders. At the health system level, reducing ACTH cost barriers and expanding vigabatrin regulatory approval in LMICs represent high‐priority targets. At the international level, development of a tiered global consensus guideline, defining minimum diagnostic and treatment standards while permitting context‐specific adaptation represents the most actionable next step toward reducing outcome disparities for children with IESS worldwide.

The strengths of this review include its global reach, inclusion of unpublished guidelines obtained directly from pediatric neurologists, and alignment with PRISMA methodology, allowing for a uniquely practice‐relevant synthesis of real‐world care. Future efforts should prioritize the development of updated international consensus guidance that harmonizes core elements of IESS management, including dosing strategies, response assessment, and monitoring protocols, while permitting context‐specific adaptations. Such guidance, informed by multinational collaboration and prospective implementation studies, will be essential to reduce global disparities, improve outcomes, and advance equitable, evidence‐based care for children with IESS worldwide.

### 4.5 Limitations

This review has several limitations. Unpublished guidelines were acquired through personal and professional networks, introducing potential selection bias. Despite efforts to improve global representation, guidelines from Sub‐Saharan Africa, Southeast Asia, and parts of the Middle East remain underrepresented (Figure 2). Institutions with greater network connectivity or academic engagement may be overrepresented, limiting generalizability to all real‐world clinical practice settings.

Additionally, the descriptive nature of this synthesis precludes causal inference or direct comparison of outcomes across treatment strategies.

As is consistent with scoping review methodology, included documents ranging from formally developed national guidelines to institutional protocols were analyzed without formal quality appraisal or differential weighting. Although national and international guidelines may carry greater evidentiary authority than institutional protocols, this approach was intentional given the review's goal of capturing the full breadth of real‐world clinical practice globally, including in settings where formal guidelines are absent.

## 5 CONCLUSIONS

This scoping review provides the first global synthesis of IESS guidelines, revealing strong consensus on core treatments (ACTH, prednisolone, and vigabatrin) but significant disparities in implementation across different resource settings. LMICs remain markedly underrepresented in published guidelines, despite bearing most of the global epilepsy burden. Given the critical importance of early treatment for neurodevelopmental outcomes, access gaps have serious consequences for children in resource‐limited settings.

Future efforts should focus on high‐quality research with standardized approaches to diagnosis and management, along with the development of new treatments for IESS. International consensus guidelines should define minimum global standards for diagnosis, treatment, and monitoring, while still allowing adaptation to local settings. Achieving this will require multinational collaboration and active involvement of clinicians across different resource settings to reduce disparities and ensure equitable care for IESS worldwide.

## AUTHOR CONTRIBUTIONS

All authors had full access to the data and take responsibility for the integrity of the data and the accuracy of the data analysis. *Study concept and design:* Gozde Erdemir. *Acquisition, analysis, or interpretation of data:* Gozde Erdemir, Anne Francine Pino, Chethan K. Rao, and Christina Briscoe. *Drafting of the manuscript:* Gozde Erdemir, Anne Francine Pino, Chethan K. Rao, and Christina Briscoe. *Figures and tables:* Gozde Erdemir and Anne Francine Pino. All other authors provided the local guidelines and translations and contributed to the final version of the article. Pediatric Epilepsy Research Consortium Infantile Spasm Work Group and International Committee members contributed to study design and methodology, facilitated international networking, provided local guidelines, and reviewed the final manuscript.

## FUNDING INFORMATION

N.S. was supported by the Italian Ministry of Health with Current Research Funds.

## CONFLICT OF INTEREST STATEMENT

R.W. has received consulting fees from Jazz Pharmaceuticals and Pendopharm. A.H. has served on an advisory board for Jazz Pharmaceuticals and as a speaker for Pendopharm Pharmaceuticals. N.S. has served on scientific advisory boards for GW Pharma, BioMarin, Arvelle, Marinus, and Takeda; has received speaker honoraria from Eisai, BioMarin, LivaNova, and Sanofi; and has served as an investigator for Zogenix, Marinus, BioMarin, UCB, and Roche. S.A. is Deputy Editor for *Epilepsia*. He has received personal fees for lectures or advice from Acadia, Angelini, Biocodex, Biogen, Camp4 Therapeutics, Encoded, GRIN Therapeutics, Jazz Pharmaceuticals, Neuraxpharm, Nutricia, Mosaica, Proveca, Servier, Stoke, UCB Pharma, Vitaflo, and Xenon. He has been an investigator for Eisai, Lundbeck, Servier, Takeda, and UCB Pharma. The authors declare no conflicts of interest. We confirm that we have read the Journal's position on issues involved in ethical publication and affirm that this report is consistent with those guidelines.

## Supporting information


**DATA S1.** Supporting information.

## Data Availability

All data relevant to this study are included in the article and supplementary materials. Additional data may be made available upon request from the corresponding author.
