# Supplementary material for: Diagnosis and management guidelines for infantile epileptic spasms syndrome around the world: A scoping review and comparative study of international approaches
Source: Epilepsia. 2026 Jun 11;67(9):4600–17. doi: 10.1002/epi.70329 (PMC13592474; doi:10.1002/epi.70329)
Supplement: Supplementary file 1 [file EPI-67-4600-s001.docx]

Supplement 1.

Search terms:

The following keywords and database‐specific terminology (e.g., MeSH) were included: 'epileptic spasm' OR 'flexor spasm' OR 'infantile spasms' OR 'infantile spasmus' OR 'spasm in flexion' OR 'west syndrome' OR 'infantile spasm' OR ‘Lightning Attacks’ OR ‘lightning attack’ OR ‘salaam attacks’ OR ‘salaam seizures’ OR ‘nodding spasm’ OR ‘nodding spams’ OR ‘hypsarrhythmia’ OR ‘hypsarrhythmias’ OR ‘infantile epileptic spasms syndrome’ OR ‘epileptic spasms’) AND ‘guidelines’ OR ‘guideline’ OR ‘guidelines as topic’ OR ‘practice guidelines’ OR ‘practice guidelines as topic’ OR ‘practice guideline’ OR ‘clinical guidelines’ OR ‘best practice’ OR ‘best practices’ OR ‘clinical guidelines as topic’ OR ‘best clinical practice’ OR ‘consensus’ OR ‘recommendations’ OR ‘position paper’ OR ‘commission’). All searches were conducted free of language or date restrictions.

Variables:

The following data was extracted from eligible guidelines: first author, publication year, geographic region, diagnostic evaluation recommendations, inpatient/outpatient EEG monitoring, duration before treatment initiation, treatment recommendations (first-line, second-line, and third-line options; sequential vs. combination therapy), response assessment timing, ketogenic diet and epilepsy surgery guidance, prednisolone, ACTH, and vigabatrin dosing protocols, side effect monitoring, vaccination schedule recommendations, and parental education content.
